# Mechanistic Perspective on C–N and C–S Bond Construction Catalyzed by Cytochrome P450 Enzymes

**DOI:** 10.1021/acsbiomedchemau.4c00100

**Published:** 2024-11-27

**Authors:** Tai-Ping Zhou, Yakun Fan, Jinyan Zhang, Binju Wang

**Affiliations:** State Key Laboratory of Physical Chemistry of Solid Surfaces and Fujian Provincial Key Laboratory of Theoretical and Computational Chemistry, College of Chemistry and Chemical Engineering, Xiamen University, Xiamen 361005, China

**Keywords:** Cytochrome P450, C−N bond, C−S bond, Natural Products, Multiscale calculations, QM/MM, MD simulations, Enzymatic mechanisms, Regioselectivity

## Abstract

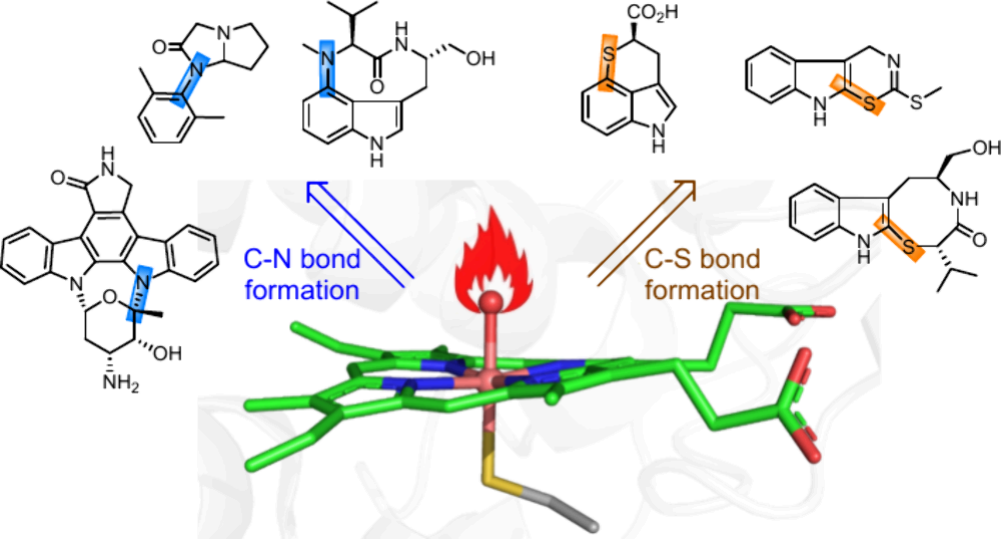

Cytochrome P450 enzymes catalyze a large number of oxidative transformations that are responsible for natural product synthesis. Recent studies have revealed their unique ability to catalyze the formation of C–N and C–S bonds, broadening their biosynthetic applications. However, the enzymatic mechanisms behind these reactions are still unclear. This review focuses on theoretical insights into the mechanisms of P450-catalyzed C–N and C–S bond formation. The key roles of the conformational dynamics of substrate radicals, influenced by the enzyme environment, in modulating selectivity and reactivity are highlighted. Understanding these reaction mechanisms offers valuable guidance for P450 enzyme engineering and the design of biosynthetic applications.

## 1 Introduction

**Figure 1:**
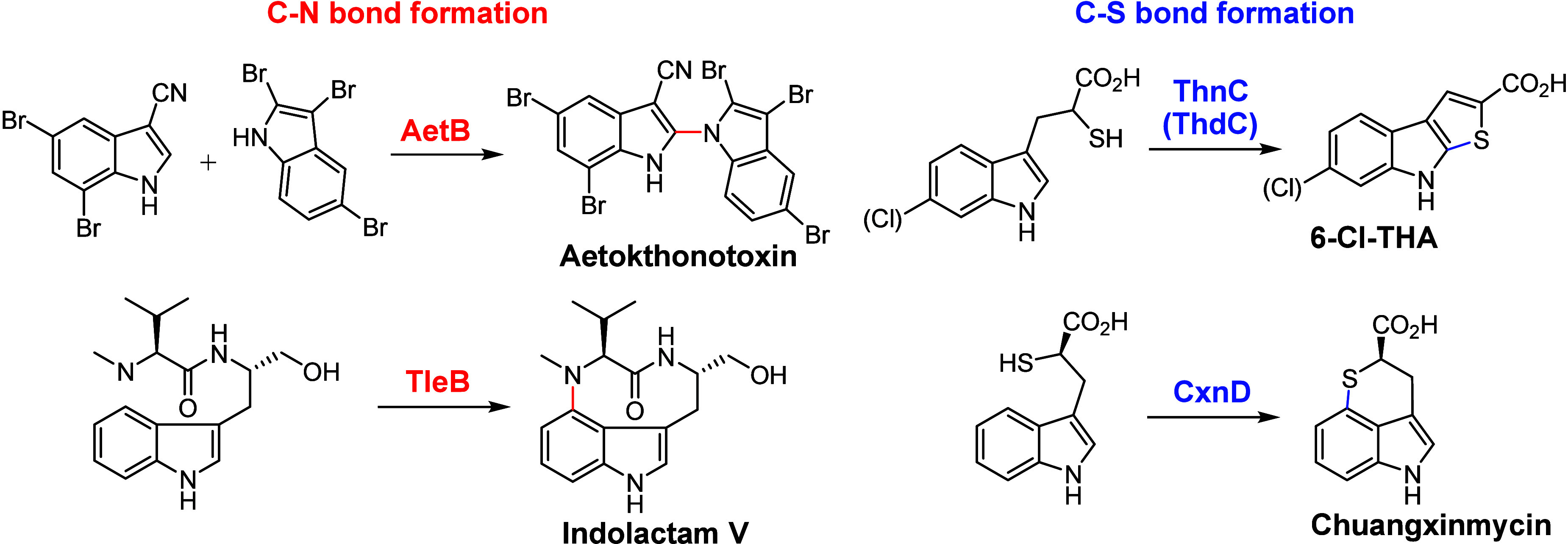
Representative C–N and C–S bond formations catalyzed by P450 enzymes.

Cytochrome P450 enzymes^1−6^ (CYP450s or P450s) catalyze a wide range of oxidative transformations in natural product (NP) synthesis,^7−12^ including polyketides (PKs),^13−15^ nonribosomal peptides (NRPs),^16−21^ alkaloids,^22−29^ and tryptophan-linked dimeric diketopiperazines^24,30−38^ (DKPs). P450 enzymes are highly ubiquitous in humans, mammals, bacteria, plants, and fungi.^39−52^ CYP450s primarily function as monooxygenases,^53−57^ catalyzing typical reactions like C–H bond hydroxylation,^58−62^ C=C bond epoxidation,^63,64^ sulfoxidation,^65^ C–O bond formation,^19,66−70^ and C–C bond formation or cleavage.^71−78^ Recent advances have revealed that P450 enzymes can catalyze a variety of C–N and C–S bond formation reactions (Figure 1), expanding the toolbox for CYP450s in synthetic applications.^79−93^ C–N or C–S bond formation can generate diverse nitrogen/sulfur-containing scaffold structures in NPs, thereby enhancing the biological functions and activities of NPs.^94−101^

Recently, extensive biochemical and structural studies have been conducted to investigate the catalytic mechanisms of P450-catalyzed C–N and C–S bond formation.^33,34,85,86,88,102^ Although crystal structures offer basic insights into substrate-enzyme interactions, the complete enzymatic reaction mechanism remains unclear based on structural analysis. For instance, the active species of Compound I (Cpd I) may favorably perform the hydrogen atom abstraction (HAA) from the substrate indole N–H in P450 TleB, since the indole N–H is in close proximity to Cpd I.^85,103^ However, the mechanism by which the enzyme active site abstracts the remote amide N–H remains unclear from the crystal structure. Furthermore, the mechanism of selective intramolecular C–N coupling in the P450 TleB remains unknown. Similar issues also occur in the NascB-catalyzed coupling of cyclo-(l-tryptophan-l-proline) to generate product (−)-naseseazine C.^33,34^

In this brief review, we focus primarily on the mechanism of P450-catalyzed C–N and C–S bond formation based on theoretical investigations.^74,104−114^ Additionally, we discuss how environmental factors modulate the selectivity and reactivity. This review emphasizes that the dynamics and positioning of substrate radical intermediates, tightly controlled by the enzyme environment, are crucial for selective C–N and C–S bond formation catalyzed by P450s. These insights are critical for advancing enzyme engineering and designing P450s for biosynthetic applications.

## 2 C–N Bond Formation

**Figure 2:**
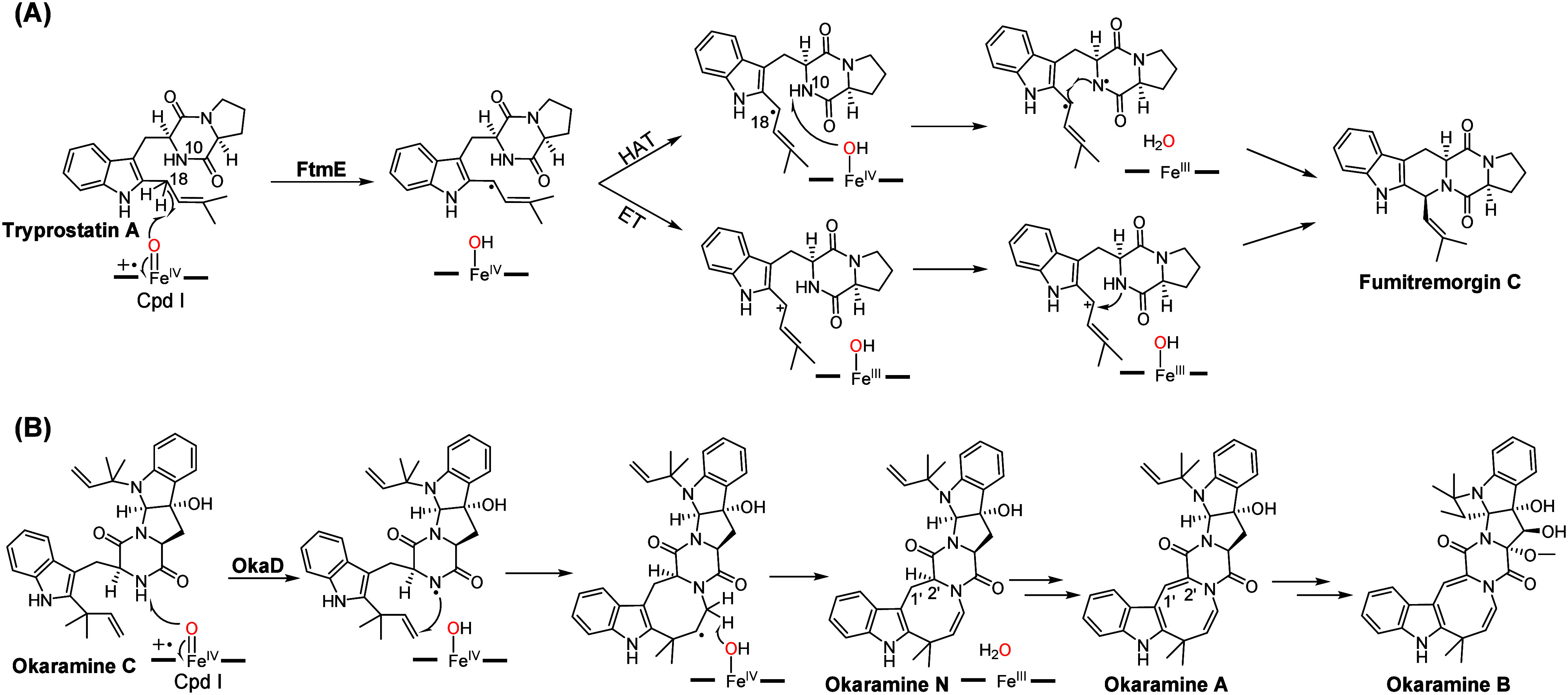
(A) Proposed intramolecular C–N bond formation mechanisms catalyzed by FtmE. (B) Proposed biosynthesis of Okaramines involving C–N bond forming catalyzed by OkaD.

**Figure 3:**
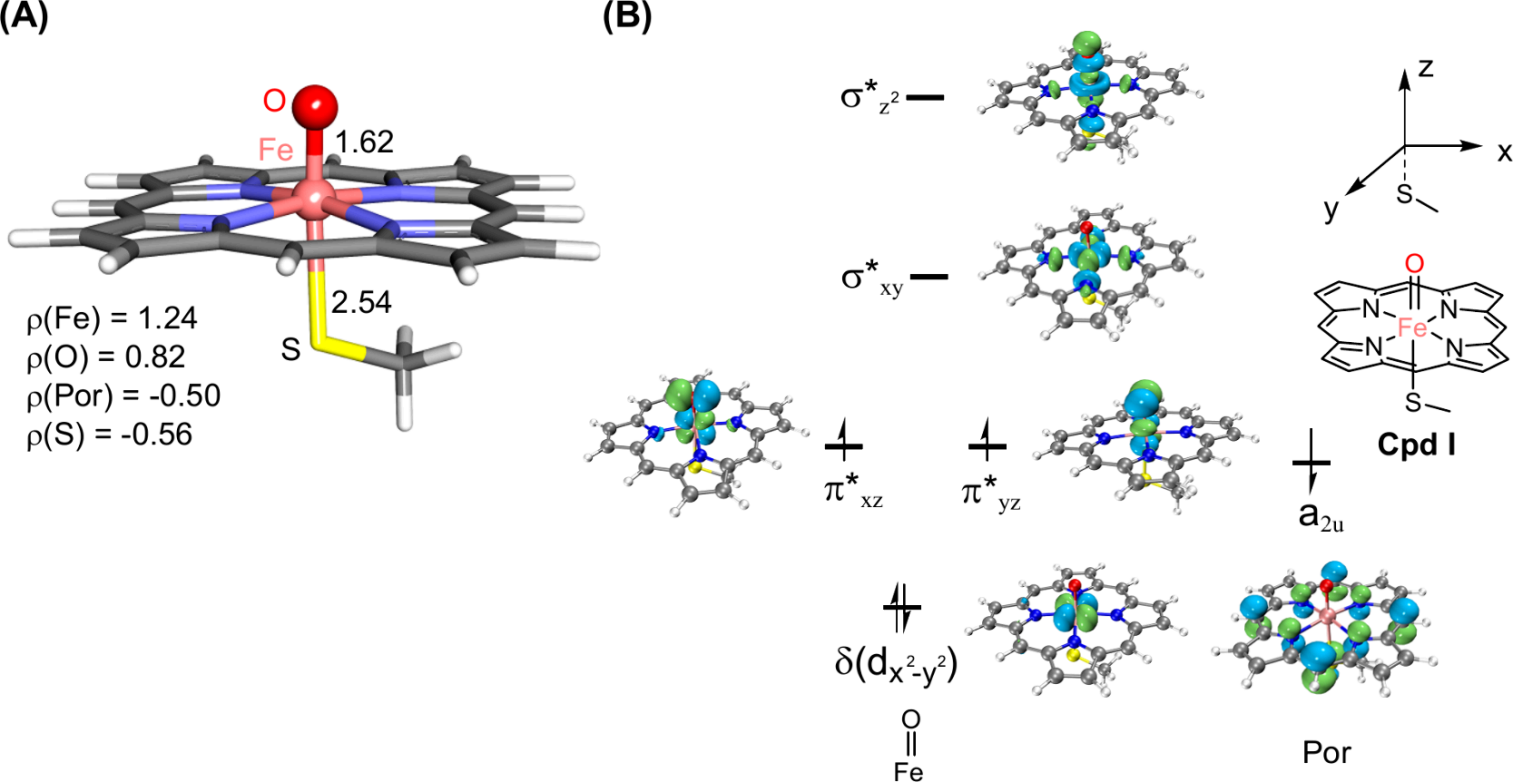
(A) QM(UB3LYP-D3BJ/def2-SVP)/MM optimized structures (Å) and group spin population data. (B) Molecular orbitals and their occupations of Cpd I species. Green and blue correspond to the positive and negative values, respectively.

Nitrogen heterocycles are among the most common motifs in NPs.^115−123^ In addition to catalyzing C–H amination through nitrene transfer reactions, P450 enzymes can facilitate both intra- and intermolecular reactions via natural catalysis.^124−128^ For example, P450 FtmE catalyzes intramolecular C–N coupling in the biosynthesis of Fumitremorgin C.^129−131^ It has been proposed that FtmE initiates the reaction through Cpd I-mediated HAA from the C18 position of Tryprostatin A, generating a substrate radical intermediate (Figure 2A). Figure 3A shows the quantum mechanical/molecular mechanical (QM/MM) optimized structure of Cpd I in a doublet. In this structure, the Fe=O and Fe–S bond distances are 1.62 and 2.54 Å, respectively.^132,133^ These bond distances generally agree with experimental structural data,^134^ supporting the reliability of the calculation method. Spin population analysis of Cpd I confirms its electronic structure as a porphyrin (Por) cation, with spin populations at Fe, O, and Por being 1.24, 0.82, and −0.50, respectively.^135^Figure 3B illustrates the orbitals and electronic configuration of Cpd I. The left-hand side displays the five d-type orbitals of iron, alongside the high-lying mixed porphyrin-thiolate orbital a_2u_. Previous QM/MM calculations have confirmed this configuration as the ground state for Cpd I species.^3,5^ Overall, both quartet and doublet spin states share the same metal orbital occupation with the difference in the spin direction of the single-occupied electron at the a_2u_ orbital. Due to their identical orbital configuration, these two spin states are close in energy.^136−138^ Two plausible mechanisms can be considered. The first involves Fe^IV^–OH mediated hydrogen atom transfer (HAT) from the N10 site, forming a diradical intermediate for C–N coupling. In the second pathway, Fe^IV^–OH may facilitate single electron transfer (ET) from the substrate radical, resulting in a substrate cation, which then undergoes C–N coupling. The OkaD-catalyzed^139,140^ C–N bond-forming cyclization is assumed to initiate with HAA from the amide N–H bond, forming an N-centered radical, which then undergoes radical addition onto the C-prenyl group (Figure 2B). This is followed by hydrogen elimination by Fe^IV^–OH, yielding Okaramine N. The mechanisms of FtmE and OkaD differ from many other P450-mediated indole alkaloid pathways, which are typically initiated by easy HAA from the indole N–H bond. This mechanistic difference suggests that the substrate may adopt a unique binding conformation, distinct from that in many other P450 enzymes.

**Figure 4:**
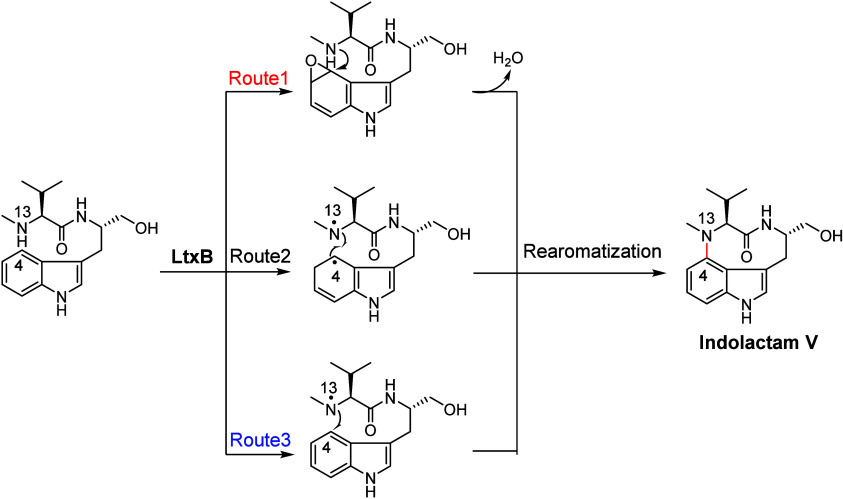
Possible mechanisms of LtxB-catalyzed C–N bond formation involved in Indolactam V biosynthesis.

Another example is the P450-catalyzed C–N bond formation in Indolactam V biosynthesis.^141^ Several P450 enzymes have been identified as catalysts for the intramolecular C–N coupling of the dipeptide *N*-methylvalyl-tryptophanol, including LtxB, TleB, and HinD.^83,102,142,143^ Early studies on LtxB suggested that the reaction may proceed through epoxidation of the indole ring, followed by nucleophilic attack by the amine group on the epoxide and rearomatization to yield Indolactam V (route 1 in Figure 4). In addition to the epoxidation pathway, two alternative pathways were proposed (routes 2 and 3 in Figure 4). Route 2 involves diradical coupling between the indole C4 radical and the valyl amide N13 radical, while route 3 involves the addition of the N13 radical to the indole ring.

**Figure 5:**
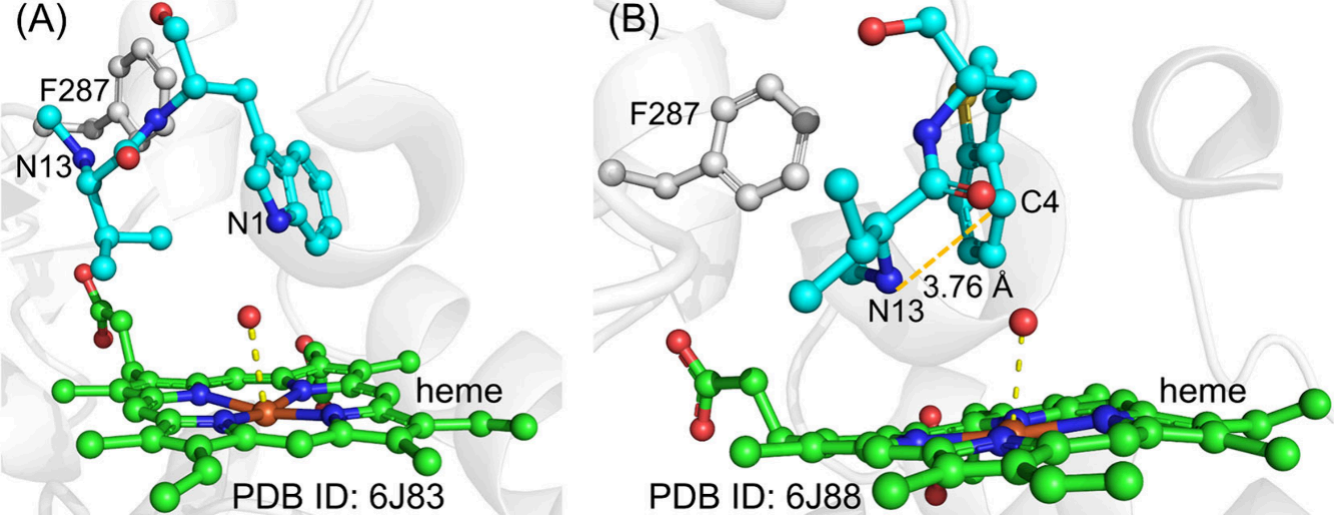
Binding modes of (A) substrate in the active sites of TleB and (B) S-substituted substrate analogue in the active sites of HinD.

Biochemical and crystallography studies have provided valuable information about the catalysis of TIeB and HinD.^85,102^ Biochemical studies indicate that the generation of a diradical at the N13 and indole N1 sites is critical for Indolactam V synthesis; supporting the diradical mechanism may be preferred. The crystal structure of TleB complexed with the native substrate (Figure 5A) reveals that the indole N1 atom is close to the heme-iron, suggesting that HAA from N1 is a likely process. This is further supported by QM/MM calculations.^105,106^ However, generating the diradical via a second HAA is not straightforward in the crystal structure, as the N13 site is distant from the heme-iron complex (Figure 5A). Interestingly, the crystal structure of HinD with substrate analogs (where indole is replaced by benzo[b]thiophene) shows that the substrate analogue can adopt an alternative conformation, with N13 positioned closest to the heme center, while the sulfur atom is distant from the heme-iron (Figure 5B). In this conformation, N13 and C4 maintain a short distance of 3.76 Å, which is suitable for C–N coupling. These findings suggest that substrate conformational changes may play a role in diradical generation and C–N coupling reactions.

**Figure 6:**
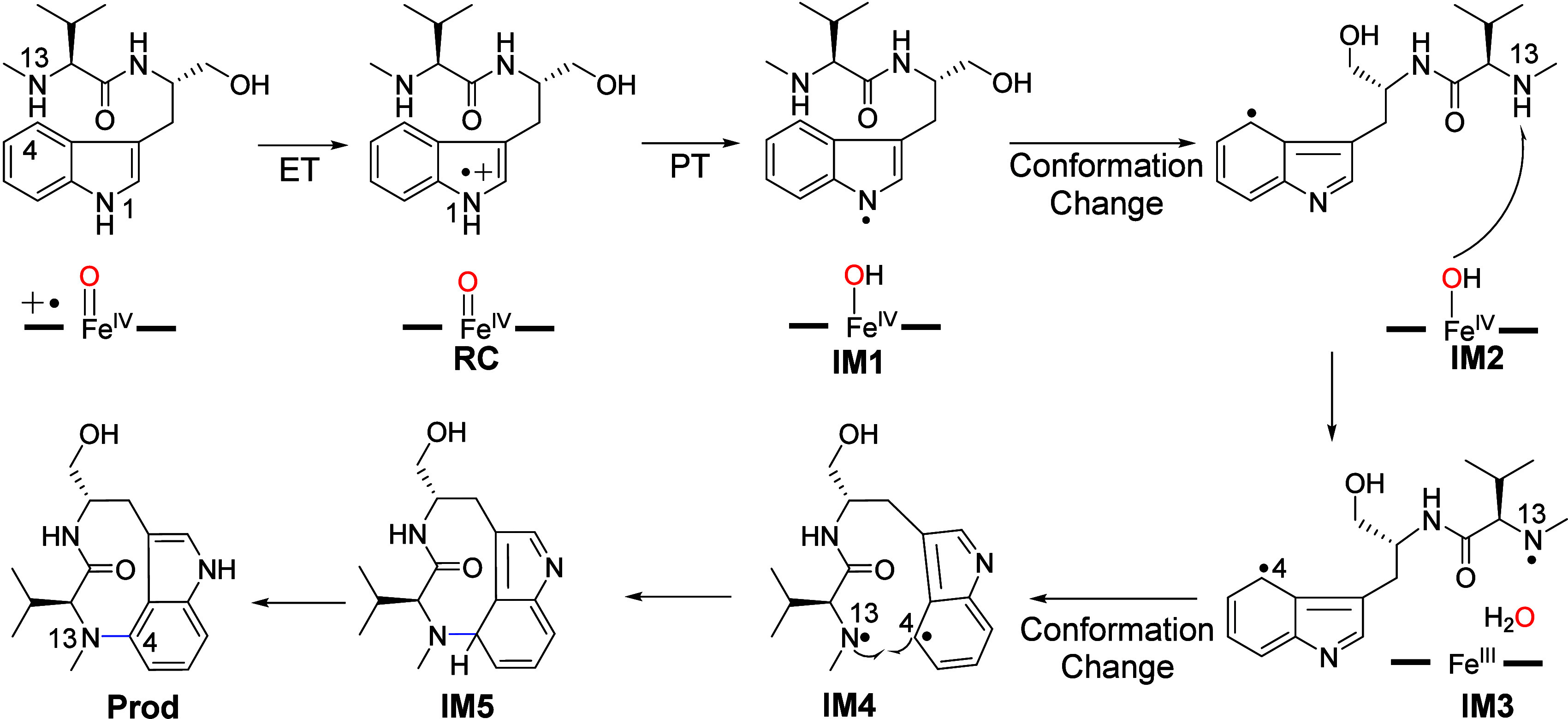
Proposed mechanism of TleB from the computational study by Liu et al. Abbreviations: PT, proton transfer.

Using the classical MD simulations and QM/MM calculations, Liu and co-workers proposed a catalytic mechanism of TleB as shown in Figure 6.^105^ In this study, QM/MM optimization of the reactant complex led to the spontaneous ET from the substrate to Cpd I, leading to Fe^IV^=O and substrate cation species (RC). Then, Fe^IV^=O can act as a base to gain the proton from the N1 site of the substrate cation, leading to the substrate radical and Fe^IV^–OH species (IM1). To facilitate the HAA from the N13 site, a manual conformational change was performed to bring the N13–H close to the Fe^IV^–OH species (IM1 → IM2). However, the second HAA (IM2 → IM3) still requires a high barrier of ∼26 kcal/mol. Moreover, the steric hindrance in IM3 is unfavorable for the subsequent C4–N13 coupling. As such, another conformational change was manually performed to bring the C4 site closer to the N13 site(IM3 → IM4), affording IM4. Starting from IM4, the C4–N13 coupling can occur to form IM5. However, the calculated barrier for the C4–N13 coupling is over 30 kcal/mol relative to RC, which is much higher than the estimated barrier of ∼19 kcal/mol for the catalytic reaction of TleB.^85^ The final rearomatization (IM5 → Prod), which can be assisted by the general acid–base catalyst in the buffer, is a facile process.

**Figure 7:**
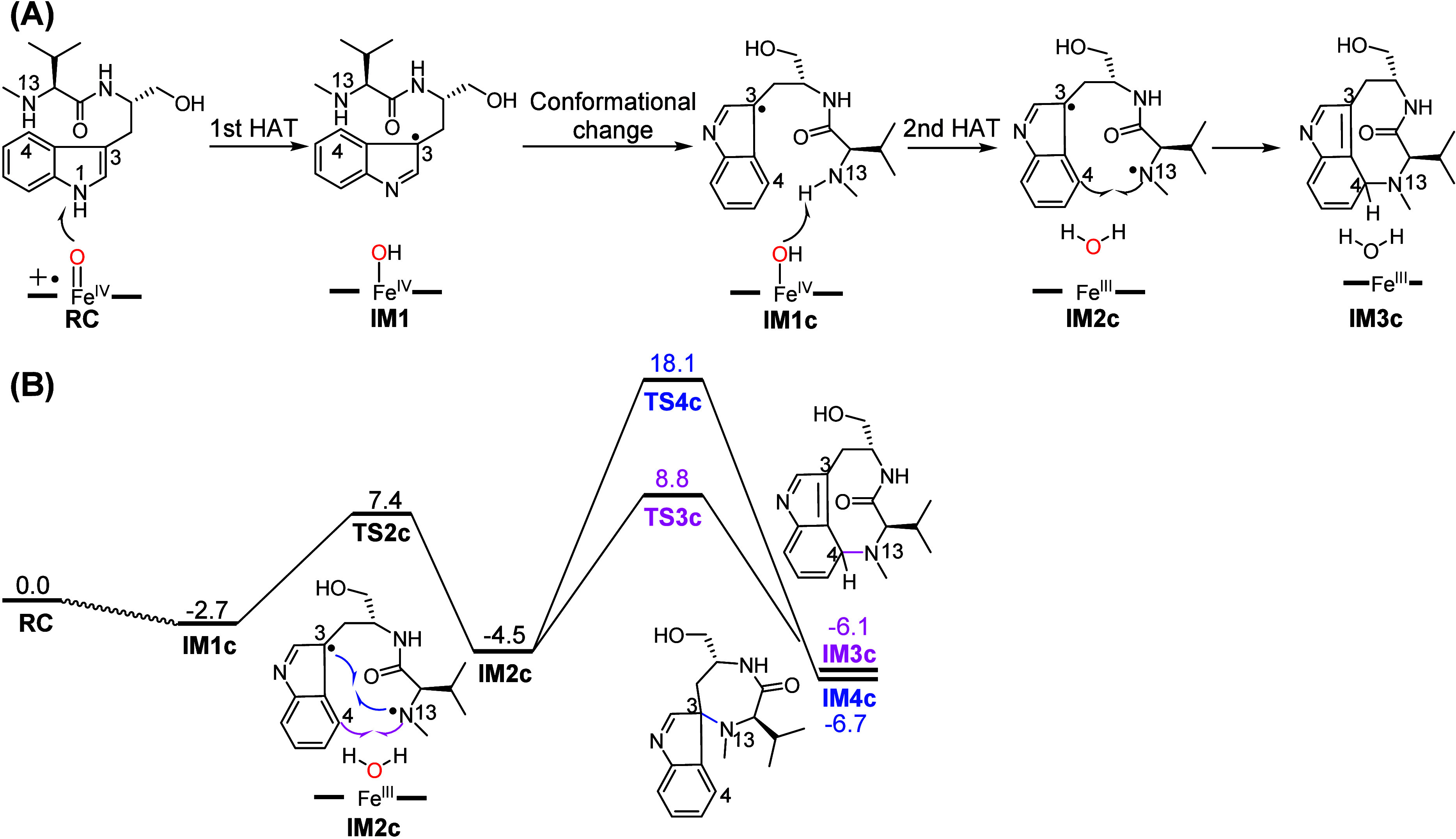
(A) Proposed mechanism of the TleB revealed in Wang’s work. (B) QM(UB3LYP-D3/def2-TZVP)/MM-calculated energy profile (in kcal/mol) for the diradical pathway after the conformational transformation from IM1 to IM1c species, along with schematic drawings of the key intermediates.

**Figure 8:**
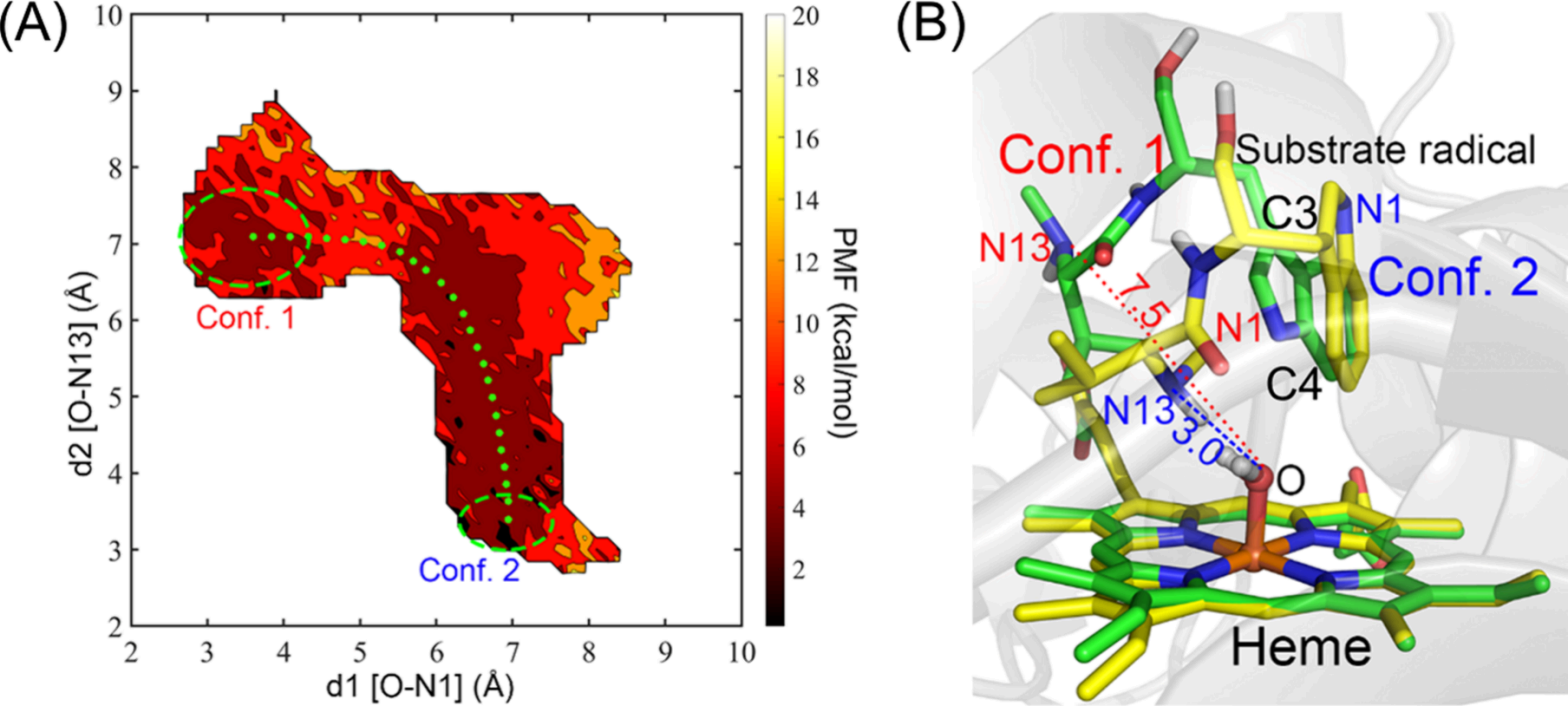
(A) Potential of mean force (PMF) profile calculated from the LiGaMD simulations. (B) Comparison between the binding conformation 1 (Conf. 1) and conformation 2 (Conf. 2) of the substrate radical. Reprinted from ref (106). Copyright 2023 American Chemical Society.

To unravel the intriguing mechanism of TleB, Wang et al. have employed multiscale computational methods, including MD simulation, QM/MM calculations, and the enhanced sampling of Ligand Gaussian Accelerated MD (LiGaMD) simulations.^106,144−146^ Unlike Liu’s study, the spontaneous electron transfers from the substrate to Cpd I were not observed in QM/MM calculations. As summarized in Figure 7A, the reaction is initiated by a facile HAT from N1–H to Cpd I, leading to the substrate radical and Fe^IV^–OH species. In line with Liu’s study, the second HAT from the N13 site and the following C–N coupling reactions are found to be unfavorable. In addition, the unwanted C–N coupling between N13 and C3 is kinetically favored over the desired C–N coupling between N13 and C4, which thus can lead to the wrong selectivity. This is logical as the indole C3 atom (∼0.6) carries much more spin population of the C3 atom than that of the C4 atom (∼0.2). As such, LiGaMD simulations have been performed to drive the conformational change of substrate radical in IM1 (labeled Conf. 1). Interestingly, an alternative binding conformation (labeled Conf. 2, see Figure 8A) can be successfully located, which has a similar energy with Conf. 1. Such conformational change of the substrate radical experiences a barrier of ∼10 kcal/mol, indicating that the process is relatively facile. In Conf. 2, the N13–H is near the Fe^IV^–OH species, while the indole N1 stays far away (Figure 8B). Interestingly, such binding conformation is analogous to the one observed substrate analogs binding conformation (Figure 5B) in homologous enzyme HinD.^102^ In addition, the demethylation reaction observed for the substrate analog further supports the conformational change of substrate during the reaction.^85^

Starting from such a new conformation, the following HAA from the N13 site is found to be facile, which involves a barrier of 10.1 kcal/mol and affords the diradical IM2c. Starting from IM2c, the subsequent C4–N13 coupling from the diradical is demonstrated to be favorable kinetically, with a barrier of 13.3 kcal/mol relative to IM2c (Figure 7B). Moreover, the C4–N13 coupling is preferred over the undesired C3–N13 coupling (with a barrier of 22.6 kcal/mol, relative to IM2c). This is mainly because the N13 site maintains a shorter distance with C4 than that with C3 in the new conformation (Figure 8B). Due to such unique positioning of the substrate, the reaction can lead to the correct regioselectivity as observed in experiments. Notably, the highest barrier of C4–N13 bond formation in the identified diradical pathway is 13.3 kcal/mol, which is lower than the barrier estimated from the experimental data. Thus, the rate-determining step of the entire catalytic cycle is expected to be the formation of Cpd I.^147^ Further analysis shows that the internal electric field (IEF) from the protein environment plays a key role in driving the conformational change of the substrate radical.^148−151^

**Figure 9:**
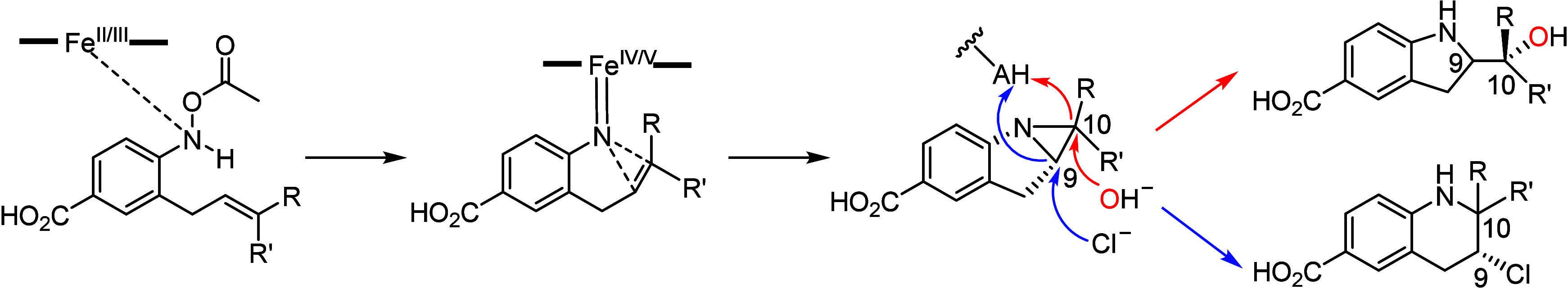
P450 BezE-catalyzed nitrenoid formation and generation of indoline and tetrahydroquinoline scaffolds.

In addition to the Cpd I-mediated radical pathway, P450s may catalyze the C–N coupling via a natural nitrene transfer reaction, as suggested for BezE^152^ (Figure 9). In this route, BezE catalyzes the elimination of acetic acid from the geranylated p-acetoxyaminobenzoic acid, the precursor for the generation of the iron nitrenoid intermediate. However, it is still unclear whether Fe^II^ or Fe^III^ is involved in the N–O cleavage reaction. Theoretically, Fe^II^ could be more reactive than Fe^III^ in catalyzing a reductive N–O cleavage, which can be learned from the Fe^II^-mediated formation of iron nitrenoid from the azide precursor in artificial P450 enzymes. Then, the reactive nitrenoid species can mediate the formation of a three-member ring intermediate, which is followed by the ring expansion reaction to synthesize indoline and tetrahydroquinoline scaffolds.

**Figure 10:**
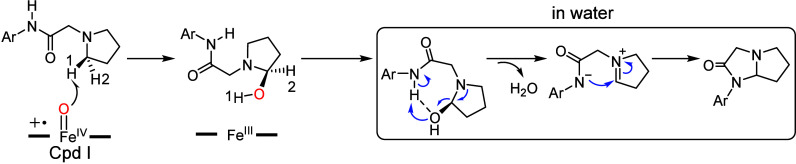
Proposed catalytic mechanism for P450-catalyzed amination of the pyrrolidine derivative of lidocaine.

In addition to the natural process mentioned above, evolved variants of cytochrome P450BM3 can catalyze the synthesis of imidazolidine-4-ones through intramolecular C–H amination.^153^ Computational studies show that the active species of Cpd I is involved in the process.^154^ As shown in Figure 10, The reaction is initiated by HAA from the H1 atom of the substrate. Interestingly, the HAA reaction is followed by spontaneous OH-rebound reaction, generating a hydroxylated intermediate. Then the hydroxylated intermediate diffused out of the enzyme active site into the water solution. Further calculations indicate that the hydroxylated intermediate readily undergoes dehydration, forming a zwitterionic intermediate, which then undergoes facile C–N cyclization to form the imidazolidine-4-one product. Notably, cyclization from the hydroxylated intermediate is much more favorable in water than in the nonpolar enzyme pocket, as the polar environment of bulk water stabilizes the zwitterionic intermediate.

**Figure 11:**
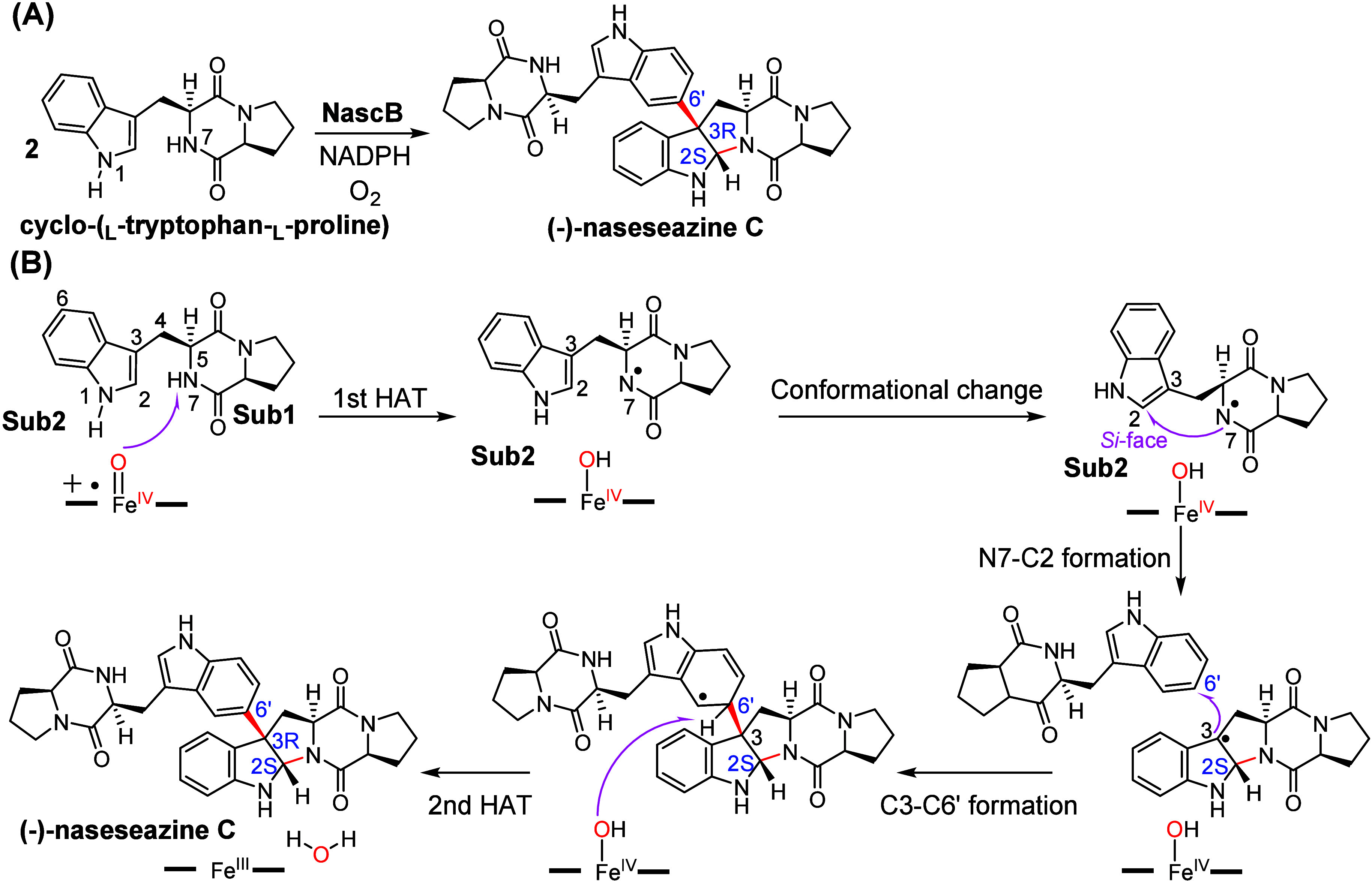
(A) Dimerization reactions catalyzed by NascB. (B) QM/MM-predicted mechanism of stereoselective dimerization by NascB.

Recently, Qu and co-workers identified the P450 enzyme NascB, responsible for the predominant formation of (−)-naseseazine C (Figure 11A).^34^ (−)-naseseazine C contains DKP scaffolds and exhibits significant structural diversity.^155−158^ However, the formation mechanism of the pyrroloindoline structure in the product remains unclear.^33,82^ Wang and co-workers conducted a QM/MM theoretical exploration of the catalytic mechanism for NascB-catalyzed coupling of cyclo-(_L_-tryptophan-_L_-proline) to generate product (−)-naseseazine C.^108^ They proposed that the reaction was initiated from the HAA from the DKP N7–H atom of substrate 1 (Sub1), resulting in the formation of a N7-centered radical species (Figure 11B). Then, a pivotal conformational change of the Sub1 radical is necessary to position the N7 atom on the *Si*-face of the indole C2=C3 double bond. Umbrella sampling was applied to investigate the “*Re* → *Si*” conformational change of the diketopiperazyl radical intermediate.^159,160^ Additional nonbonding interaction analysis supports that both conformations of the radical species can be stabilized by the protein environment. This is followed by intramolecular C2–N7 bond formation to generate the right configuration pyrroloindoline radical species intermediate. The subsequent intermolecular C3–C6’ bond formation was found to occur via a radical-mediated mechanism, instead of a cationic Friedel–Crafts mechanism. Moreover, the conformational switch of the Sub1 radical not only lowers the barrier of the intermolecular C3–C6’ bond formation but also affords the correct stereoselectivity as observed in experiments. Based on the computational study, the rate-determining step of the overall enzymatic is the C3–C6’ formation with a barrier of 23.0 kcal/mol. Their study highlights that the conformational movement of the substrate, which is controlled by the protein environment, can be vital to the P450-catalyzed dimerization reactions.

**Figure 12:**
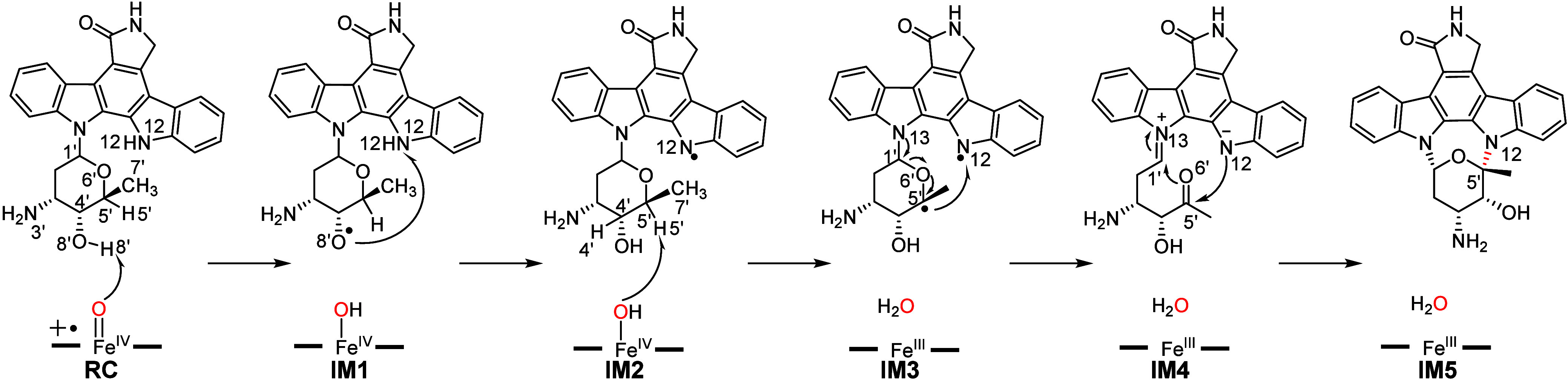
Proposed catalytic mechanism of C–N coupling by SpcN.

Very recently, Li and co-workers identified the P450 enzyme SpcN, which catalyzes an unusual *sp*^3^ C–N bond formation during the biosynthesis of staurosporine.^96^ They proposed the catalytic mechanism of SpcN based on crystal structure analysis and computational studies (Figure 12). The reaction is initiated with HAA from the substrate O8’–H8’ bond by Cpd I, forming the O8′-centered radical IM1. In the nascent IM1, the O8’ radical abstracts a hydrogen atom from the N12–H12 bond, yielding the more stable N12-centered radical intermediate IM2. From IM2, the Fe^IV^–OH abstracts another hydrogen atom from C5′–H5′ to form diradical species IM3. Notably, the resulting diradical species is highly unstable, leading to the rapid cleavage of the C1’–O6’ bond and the formation of an unprecedented zwitterionic intermediate, differing from the diradical mechanism observed in other P450 enzymes. Natural bond orbital (NBO) analysis further supported the formation of a double bond between N13 and C1’ in IM4.^161,162^ Finally, the negatively charged N12 nucleophilically attacks the C5′ site, coupled with C1’–O6’ bond formation and charge rearrangement, producing staurosporine. According to their calculations, the HAA from substrate C5′–H5′ is the rate-determining step with an overall barrier of 20.3 kcal/mol, which is in good agreement with the experimental kinetics. To further validate the proposed mechanism, MD simulations were performed with the 3′-*N*-acetyl substrate, which could not be catalyzed by SpcN. MD simulation results show that the introduced acetyl group hinders the approach of the O8’–H8 bond to Cpd I. Both experimental and simulation results of the substrate analogue support the proposed SpcN-catalyzed mechanism. Their study emphasizes that the conformational movement of the substrate, regulated by the protein environment, is crucial for P450-catalyzed dimerization reactions. This mechanistic view of SpcN differs from the widely recognized diradical mechanism in other P450 enzymes that catalyze C–N bond formation. It offers a new theoretical perspective on P450-catalyzed C–N bond coupling.

## 3 C–S Bond Formation

**Figure 13:**
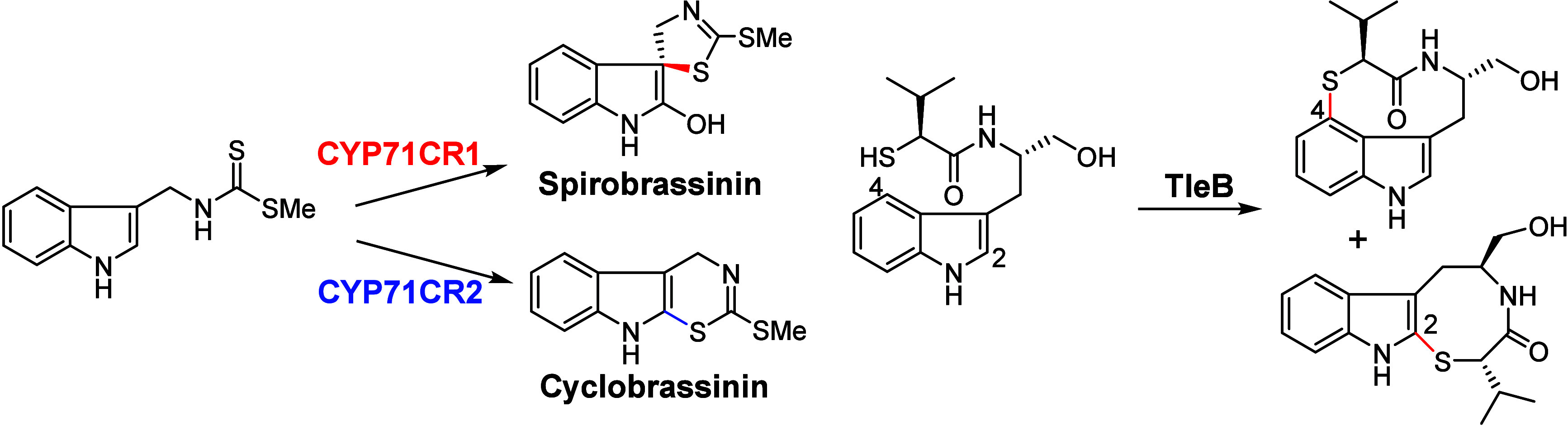
P450-catalyzed C–S bond formation.

Similar to nitrogen heterocycles, sulfur heterocycles are also significant motifs found in NPs.^58,163−165^ The enzymatic formation of carbon–sulfur bond has been comprehensively reviewed before.^166^ This section primarily focuses on the mechanisms of P450-catalyzed C–S bond formation. As illustrated in Figures 1 and 13, several P450 enzymes have been identified to catalyze the intramolecular C–S bond formation from indole derivatives.^167−170^ Two P450 enzymes, CYP71CR1 and CYP71CR2, from *Brassica rapa* (Chinese cabbage) have been characterized as catalysts for intramolecular C–S bond formation in the biosynthesis of Spirobrassinin and Cyclobrassinin (Figure 13).^171^ However, these two P450 enzymes exhibit different regioselectivities in the cyclization reaction. P450 ThnC catalyzes C–S bond formation from the 6-Cl-thiotryptophan substrate, producing a tricyclic indole S-hetero scaffold^90,170,172^ (Figure 1). TleB, initially characterized as a catalyst for C–N bond formation in Indolactam V biosynthesis, was found to also catalyze intramolecular C–S bond formation from a thiol-substituted substrate.^86^ However, TleB exhibits loose control over regioselectivity, yielding a mixture of S–C4 and S–C2 linked products (Figure 13). CxnD, which shares a similar structure with TleB, catalyzes C–S bond formation in Chuangxinmycin biosynthesis^88,91^ (Figure 1).

**Figure 14:**
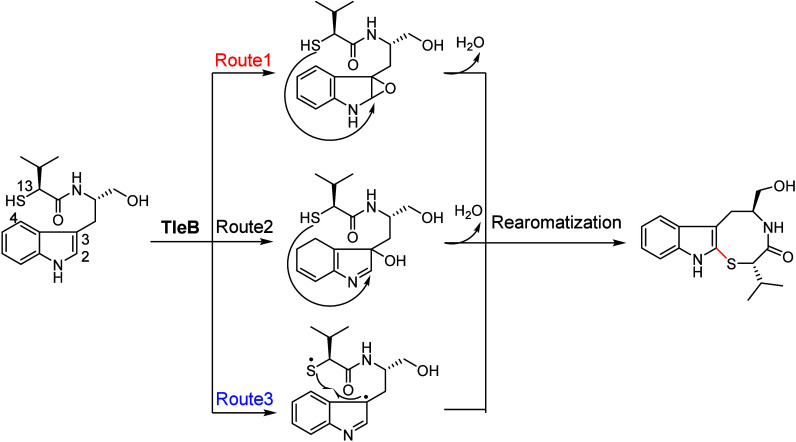
Possible mechanisms of P450-catalyzed C–S bond formation involved in sulfur-containing indolactam derivative biosynthesis.

**Figure 15:**
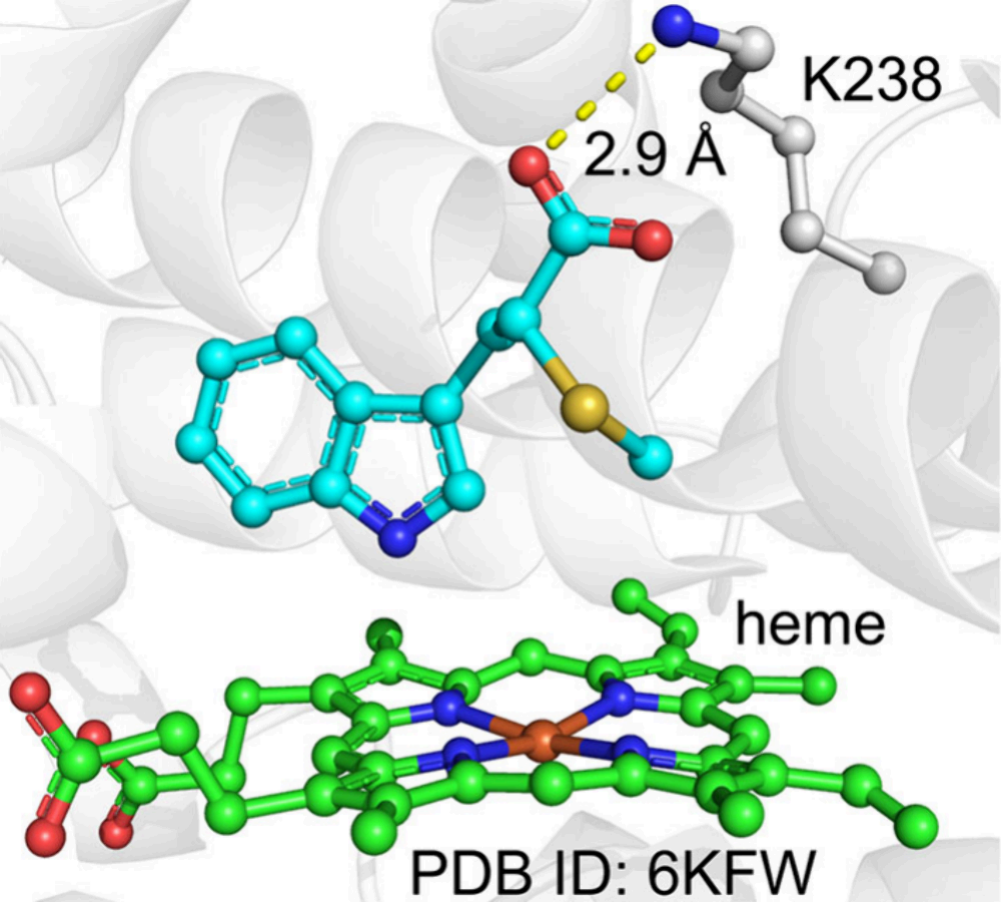
Active site structure of CxnD in complex with the substrate analog.

Three mechanistic routes (Figure 14) can be proposed for the P450-catalyzed C–S bond formation mechanism.^86^ The first route involves the oxidation of the C2=C3 double bond of indole to form an epoxide intermediate, followed by a nucleophilic attack from the thiol to generate a hydroxylated *S*-heterocyclization intermediate. In the second route, the radical generated after the first HAT from indole N–H translocates to the C3 position and undergoes OH-rebound to form a hydroxylated intermediate. Subsequently, an intramolecular attack by thiol on the indole generates the cyclized intermediate. The subsequent dehydration generates the S–C2 linked product. However, the concerted epoxide-ring opening, C–S bond formation, and H-shift are expected to be energetically demanding. Additionally, the reported X-ray structure indicates that the indole moiety of the substrate analog adopts an upright conformation, which is unfavorable for epoxidation by the Cpd I species (Figure 15). Furthermore, the barrier for OH rebound to the C3 site, which is highly sensitive to substrate positioning, remains significant. In an alternative mechanism, the reaction may occur via a diradical mechanism similar to TleB-catalyzed C–N formation.^85^ In this mechanism, the reaction can be initiated by HAA from the N1 site of indole moiety, as inferred from the reported X-ray structure.^88^ The substrate radical may then undergo a conformational change to bring the S–H moiety close to the Fe^IV^–OH species.

**Figure 16:**
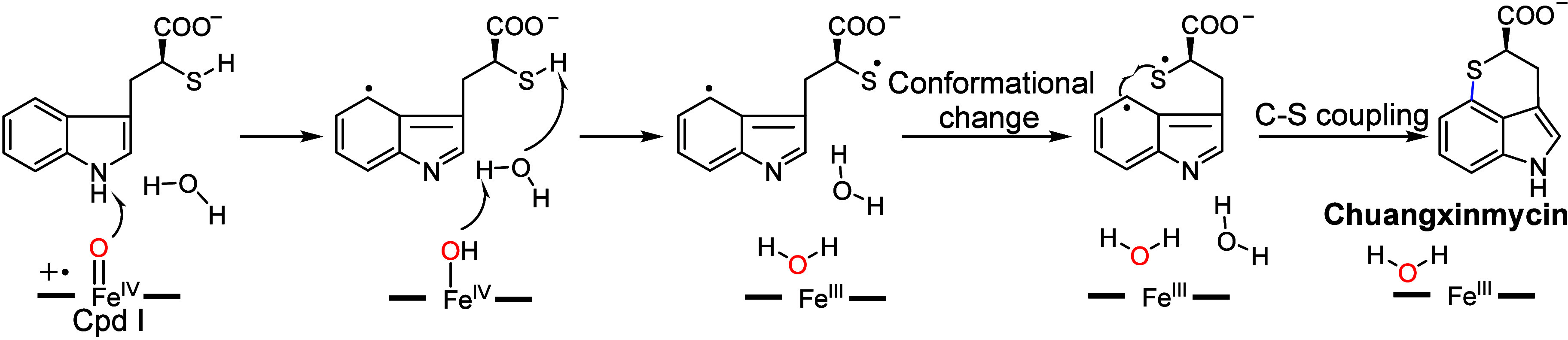
Proposed mechanism for CxnD based on the calculations.

Recently, Liu and co-workers investigated the CxnD-catalyzed intramolecular C–S coupling in the biosynthesis of Chuangxinmycin.^107^ Based on MD and QM/MM simulations, a water-assisted mechanism was proposed (Figure 16). The reaction is initiated by HAT from substrate N–H to Cpd I, leading to the substrate radical. Then, solvent water located between substrate S–H and Fe^IV^–OH can assist the second HAT reaction, possibly via a proton-coupled electron transfer (PCET) mechanism.^19,173−176^ To facilitate the following C–S coupling, the diradical intermediate must undergo a conformational change, from which two radical centers can approach each other. The subsequent C–S coupling and proton relocation can generate the final product. Notably, the QM/MM predicted energy profiles show that the proposed mechanism initiated by the HAT from substrate N–H has a comparable overall barrier as the one initiated by the HAT from substrate S–H, making these two pathways kinetically indistinguishable.

**Figure 17:**
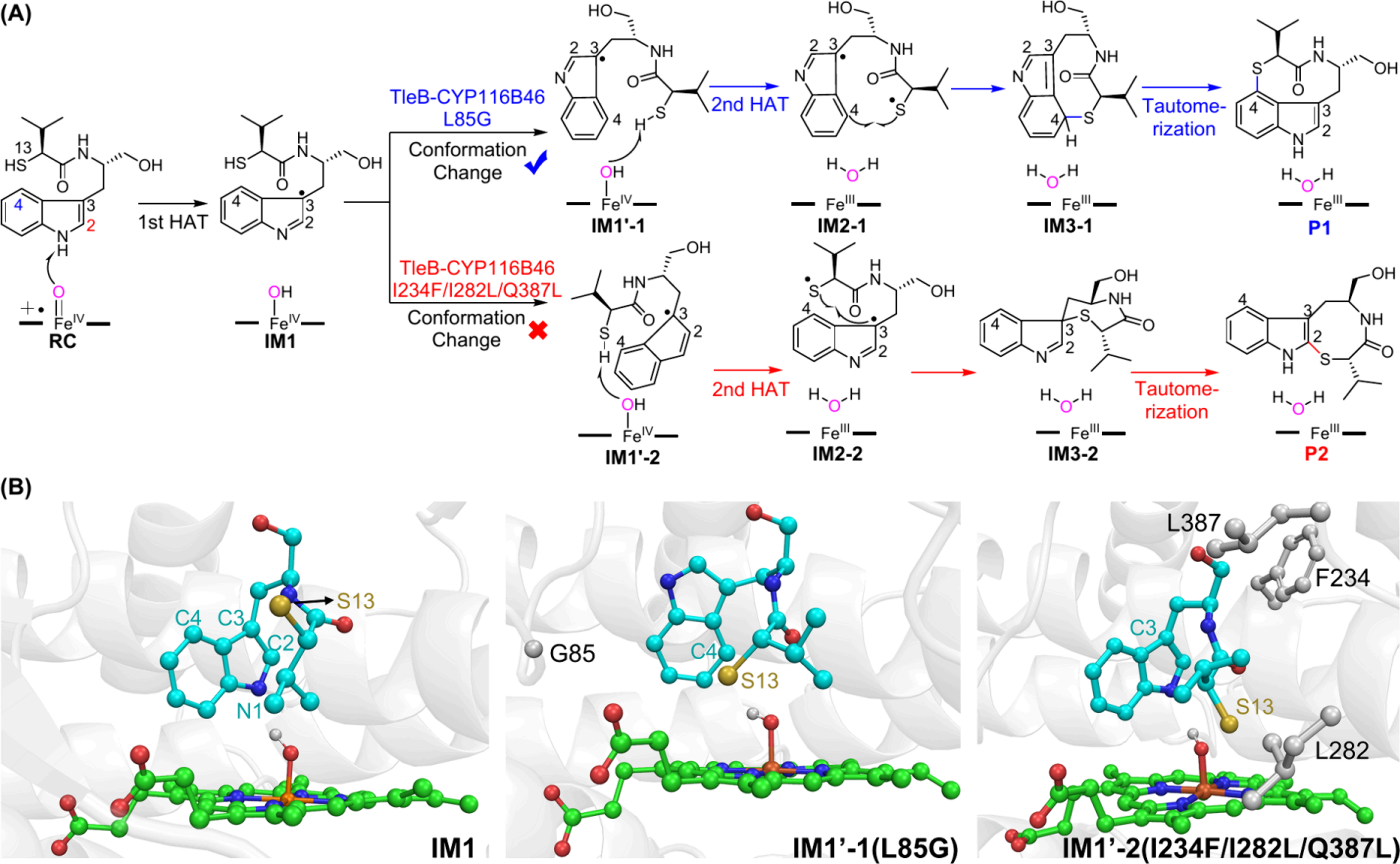
(A) Proposed mechanism for two TleB variants catalyzed C–S bond formation from 13-thiol substituted substrate based on the calculations. (B) Structures of key species involved in the reaction.

Very recently, Zhao and co-workers investigated the molecular mechanism of the P450 TleB-catalyzed selective C–S bond formation reaction through extensive simulations (Figure 17A).^89^ The reaction is demonstrated to proceed through a diradical coupling mechanism that is analogous to the P450 TleB-catalyzed C–N formation (Figure 7A). It was found that the dynamics and positioning of the radical species, which is well controlled by the surrounding residues, are key to the selective C–S bond formation reaction. As shown in Figure 17B, the L85G variant of TleB can facilitate the conformational switch of the indole radical group in IM1, affording to IM1′-1, in which the C4 site of the substrate is close to the S13 atom, thereby leading to the selective C4–S13 bond formation for the P1 product. By contrast, the I234F/I282L/Q387L variant barricades the conformational switch of the indole radical group in IM1, resulting in C3–S13-linked intermediate IM3–2. Afterward, IM3–2 can isomerize into the more stable product of P2 in a water solution. This study unveils the molecular basis of selective C–S coupling by P450 TleB and presents a promising approach for achieving high-purity biocatalytic synthesis of valuable sulfur-containing heterocyclic products.

## 4 Summary and Outlook

In this review, we present a series of compelling mechanisms for C–N and C–S bond formation catalyzed by CYP450s. Insights from theoretical studies underscore the critical role of the enzyme environment in influencing the selectivity and reactivity of P450-catalyzed C–N and C–S bond formation. P450-catalyzed C–N and C–S bond reaction usually begins with H-abstraction from the weak N–H or S–H bond by Cpd I. To facilitate the following C–N or C–S bond coupling, the radical intermediate may require a conformational change within the active site. The substrate radical intermediates’ dynamics and positioning, which have been modulated by the enzyme environment, enable selective C–N and C–S bond formation in P450s. A thorough understanding of these mechanisms enables researchers to engineer P450 enzymes with tailored functionalities for specific biosynthetic applications. In addition to C–N and C–S formation, P450-catalyzed C–O and C–C bond formation reactions are extensively involved in the biosynthesis of NPs.^66,69,78,177,178^ Computational studies also support a diradical mechanism for P450-catalyzed C–O and C–C bond formation.^19,178−180^ Additionally, it was found that some P450s also require conformational changes of the substrate to realize C–C coupling.^180,181^ This mechanistic understanding will enhance our ability to predict and manipulate enzyme behavior, ultimately contributing to the efficient and selective synthesis of diverse NPs.
